# Printability of alloys for additive manufacturing

**DOI:** 10.1038/srep19717

**Published:** 2016-01-22

**Authors:** T. Mukherjee, J. S. Zuback, A. De, T. DebRoy

**Affiliations:** 1 Department of Materials Science and Engineering, The Pennsylvania State University, University Park, PA 16802, United States

## Abstract

Although additive manufacturing (AM), or three dimensional (3D) printing, provides significant advantages over existing manufacturing techniques, metallic parts produced by AM are susceptible to distortion, lack of fusion defects and compositional changes. Here we show that the printability, or the ability of an alloy to avoid these defects, can be examined by developing and testing appropriate theories. A theoretical scaling analysis is used to test vulnerability of various alloys to thermal distortion. A theoretical kinetic model is used to examine predisposition of different alloys to AM induced compositional changes. A well-tested numerical heat transfer and fluid flow model is used to compare susceptibilities of various alloys to lack of fusion defects. These results are tested and validated with independent experimental data. The findings presented in this paper are aimed at achieving distortion free, compositionally sound and well bonded metallic parts.

Additive manufacturing (AM) allows for one-step fabrication of complex parts that are true to their designs. It eliminates the need for assembling multiple components, training a new workforce or setting up any new equipment while simultaneously minimizing manufacturing time and wastage of materials and energy. It is projected to become a 16 billion dollar industry over the next five years^1^. Its current applications include printing of tissues^2^,^3^, implants and prosthesis^4^, electronics^5^, aero-engine components, compositionally graded parts^6^ and corrosion resistant protective coatings^7^. Although AM is rapidly growing to produce metallic, polymeric and ceramic components, production of metallic parts is its fastest growing sector.

In order to successfully print a metallic part, an appropriate alloy must be selected. Parts should be dimensionally accurate, the chemical composition of the final product should be the same as that of the alloy powder and successive layers need to be adequately bonded by fusion. An understanding of printability, or the ability of an alloy to resist distortion, compositional changes and lack of fusion defects, is essential for both powder injection and powder bed based AM processes. What are needed and not currently available are quantitative scales to construct, test and validate the printability of different alloys in these processes.

During AM of metal parts, alloys undergo spatially-variable heating, melting, solidification and cooling of the entire part. Permanent deformation in various regions of a part can occur depending on thermo-physical properties of the alloy, the rigidity of the part and transient temperature fields^7^. An appropriate model for the estimation of strain can provide an assessment of the susceptibility of various alloys to distortion and the resulting dimensional inaccuracy of the final part in a quantitative scale.

Most engineering alloys contain multiple alloying elements that vaporize rapidly at high temperatures and can be selectively lost during AM. Consequently, the chemical composition of the part may be different from that of the original material. For all alloys, a reduction in peak temperature and a smaller surface-to-volume ratio of the molten pool will minimize pronounced changes of chemical composition during laser processing^8^,^9^.

Lack of fusion defects originate from inadequate penetration of the molten pool into the substrate or previously deposited layer. Important variables include thermo-physical properties, characteristics of the heat source and processing parameters that determine the geometry of the melted region. Satisfactory penetration of the molten pool into the substrate or the previously deposited layer needs to be ensured to avoid this defect.

Here we report the development of theories to examine the propensities of common alloys to form the three most common defects during powder-based AM. These theories are tested using available independent experimental data. The methodology and results presented here are aimed at providing a quantitative basis for overcoming common defects in the AM of metallic parts.

## Heat transfer and fluid flow modeling

Dimensional inaccuracy, loss of alloying elements due to vaporization and lack of fusion defects encountered during AM of metal parts depend on the geometry of the molten pool and temperature distribution. However, real time measurement of these quantities during AM is difficult. Therefore, a well-tested, 3D transient heat transfer and fluid flow model is used to calculate these quantities. The model solves equations of conservations of mass, momentum and energy to provide 3D transient temperature and velocity fields as well as the shape and size of molten pool. The methodology is well documented in the literature^10^ and its implementation for an AM process has been described in the supplementary information.

For simplicity, a flat molten pool surface is assumed in the calculations. The flat surface assumption gives comparable values for pool dimensions as compared to free surface model. For example, Ha *et al.*^11^ showed that the flat surface assumption resulted in about 3% difference in molten pool width and depth compared with the free surface model. It is estimated that the errors in strain owing to flat surface assumption is about 3%. An analysis of the error in estimation of thermal strain owing to flat surface assumption is included in the supplementary document.

**Figure 1:**
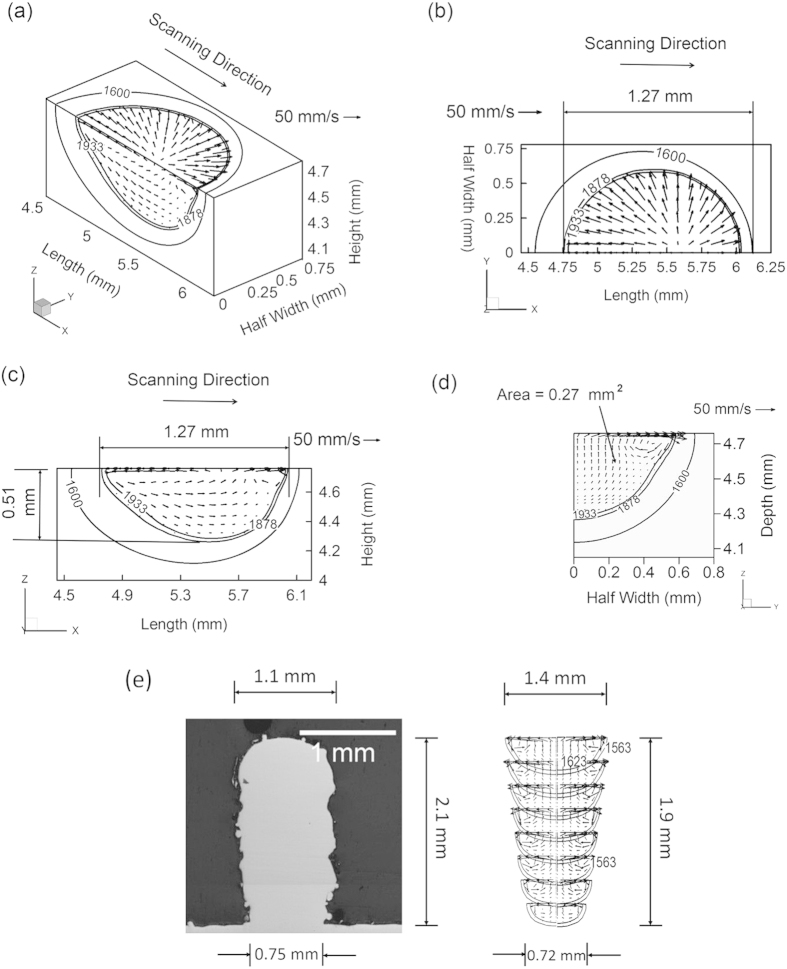
Computed 3D transient temperature and velocity field in laser based AM process using Parameter Set 1 in Table 1 for second layer deposition of Ti-6Al-4V (**a**) Isometric view where 1933 K and 1878 K represent liquidus and solidus temperatures, respectively. The isotherm of 1878 K represents the molten pool boundary. Half of the pool is shown due to the symmetric nature of the pool about the x–z plane. A reference velocity vector of 50 cm/s has been shown to comprehend the liquid metal flow velocity in the molten pool. (**b**) Measurement of the length of the pool on top view. (**c**) Measurement of depth of penetration of the pool on longitudinal cross-sectional view. (**d**) Transverse cross-sectional view to measure the area of the pool perpendicular to the scanning direction using Parameter Set 2 in Table 1 for IN625 (**e**) Pool shape and size comparison for single track 8 layers deposition between numerically calculated pool and experimentally obtained pool taken from literature^12^.

Figure 1(a–d) show the 3D temperature and velocity field in a laser based AM process for Ti-6Al-4V during the deposition. Figure 1(e) shows that the calculated build shape and size are in fair agreement with experimental results^12^. The agreement between the computed and the experimental results indicates that the model can be used to estimate thermal strains, composition changes and lack of fusion defects with confidence.

### Dimensional inaccuracy

Dimensional inaccuracy in AM parts due to thermal distortion is caused by non-uniform expansion and contraction of different regions of the part that experience changes in temperature. Thermal distortion during the deposition process depends on alloy properties, heat input, deposition time, substrate dimensions, part geometry, time delay between the deposition of successive layers and other variables. Propensity for thermal distortion is calculated from the maximum thermal strain. Using experimental data and dimensional analysis, the maximum thermal strain is estimated as a function of these important variables.[Table t1]

A non-dimensional thermal strain parameter (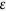
*) is used to represent the maximum thermal strain. A relation between this parameter (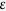
*) and the AM variables is developed based on the Buckingham π-theorem^13^. Table 2 provides a list of these variables along with their dimensions in MLTθ system. Since there are 4 fundamental dimensions and 8 variables, there are four (8 – 4 = 4) π terms. Non-repeating variables are chosen to be *ρV*, *h*, Δ*T* and *k/C_P_*. Applying Buckingham π-theorem, the final four π terms can be written as


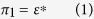



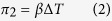



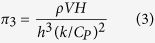



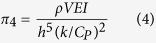


From the above relationships, the thermal strain parameter can be expressed as a function of the AM variables as:


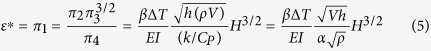


The heat transfer in AM processes is transient in nature which is best characterized by the Fourier number (*F*) given as 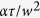 where *α*, *τ* and *w* refer to thermal diffusivity, characteristic time scale and length through which the heat conduction occurs, respectively. The Fourier number (*F*) can be rewritten as 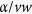 considering *v* as the beam scanning speed and *w* as the length of the molten pool. The term 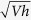 in equation (5) is dimensionally equivalent to *w*^2^. Therefore, the thermal strain parameter, 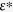, can be expressed in terms of the Fourier number (*F*) as


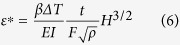


Equation (6) indicates that high strains result from large volumetric change (*βΔT*), long deposition time (*t*) and high rates of heat input per unit length (*H*). In contrast, terms in the denominator of equation (6) indicate factors that are helpful to reduce thermal strain. For example, a high flexural rigidity (*EI*) of a structure resists deformation. Similarly, a high Fourier number (*F*) indicates faster diffusive heat transfer relative to heat accumulation and a high rate of heat transfer reduces the peak temperature and thus, the thermal strain.

**Figure 2:**
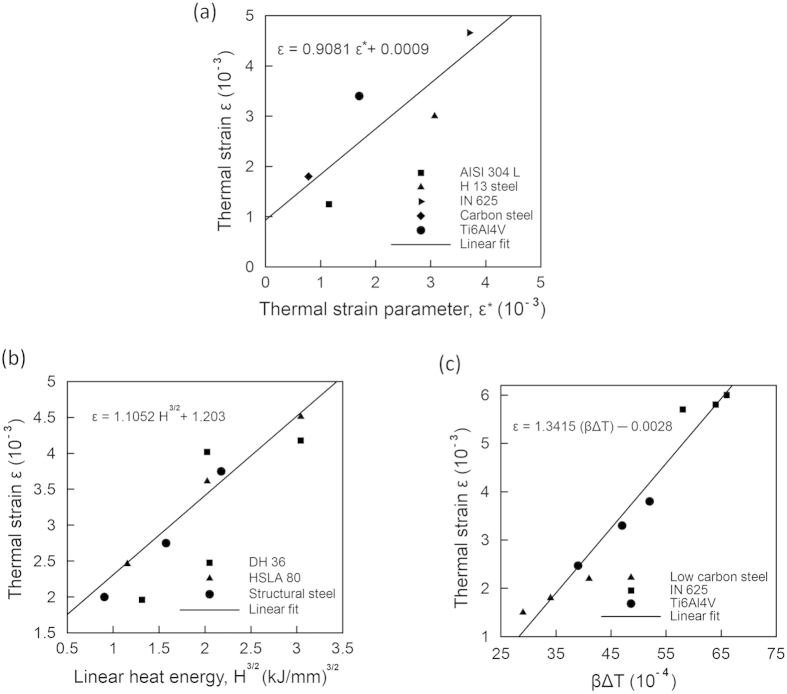
Values of maximum thermal strain ε (**a**) as a function of the thermal strain parameter for five alloys^14^,^15^,^16^,^17^,^18^ showing a linear relationship (**b**) as a function of H^3/2^ for structural steel^19^, tool steel^20^ and high strength low alloys steel^20^ in welding (**c**) as a function of βΔT for low carbon steel^15^, IN 625^14^ and Ti-6Al-4V^14^ in AM.

Figure 2(a) shows the maximum thermal strains 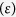 obtained from the experimentally measured thermal distortions^14^,^15^,^16^,^17^,^18^ as a function of the thermal strain parameter (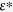), which is estimated using equation (6). A sample calculation for the estimation of the thermal strain parameter (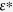) for Ti-6Al-4V is shown in the supplementary document. Figure 2(a) indicates that the maximum thermal strain (*ε*) for an AM part can be expressed as a linear function of the thermal strain parameter (ε*). Based on the trend of the data points presented in Fig. 2(a), the maximum thermal strain (*ε*) can be expressed as


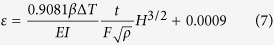


Heat input per unit length (*H*) and volumetric change (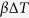) are two important variables in AM and equation (7) shows that these affect the maximum thermal strain (*ε*) in direct proportion. Equation (7) is validated by examining the effects of heat input and volumetric change on the maximum thermal strain using independent experimental data^14^,^15^,^19^,^20^. Figure 2(b,c) show that both *H*^3/2^ and *βΔT* influence thermal strain linearly, regardless of the alloy. The linearity of the plots is indicated by the correlation coefficients of 0.92 and 0.98 for Fig. 2(b,c), respectively. Figure 2(a) through 2(c) show that equation (7) can estimate the maximum thermal strain (*ε*) to provide a direct measure of the expected thermal distortion. Equation (7) is valid for the heat input range of 150–2800 J/mm, which is widely used for major AM applications^7^,^21^. Figure 2(a) presents independent experimental data which includes strains due to phase transformations. Tool paths during AM can have significant effects on thermal distortion. Wei *et al.*^22^ showed that the heat transfer pattern and pool dimensions change depending on the tool path. Therefore, the Fourier number also depends on the tool path, which is accounted for in equation (7).

**Figure 3:**
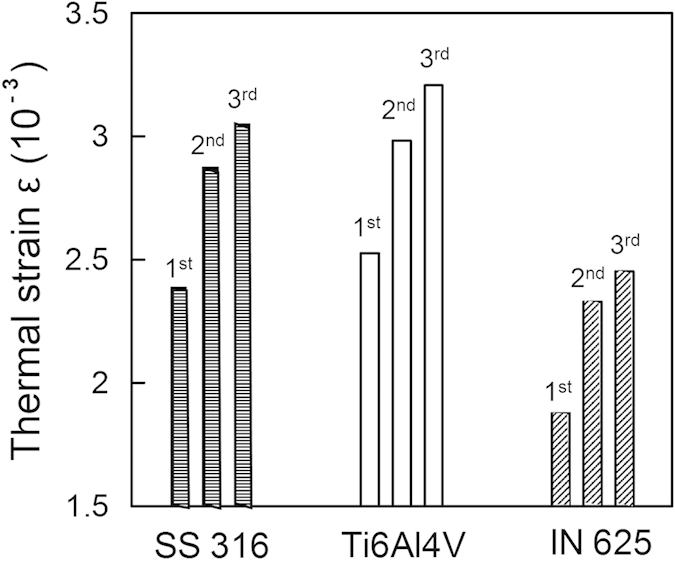
Values of maximum thermal strain ε in laser additive manufacturing (LAM) of a single-track three-layer deposition of SS 316, Ti6Al4V and IN 625 powder materials using Parameter Set 1 in Table 1.

Equation (7) provides a usable scale to estimate and compare the maximum thermal strain in laser-based AM for different alloys. A relatively high value of thermal strain calculated using equation (7) signifies more thermal distortion and a lower printability of the corresponding alloy. Figure 3 shows that increasing the number of layers increases thermal strain. This is caused by lower heat conduction from the molten pool into the substrate resulting in higher temperature difference (*∆T*). Thermal strain is the highest for Ti-6Al-4V, which can be attributed to its relatively low density and thermal diffusivity. The ranking of the alloys in Fig. 3 provides a relative scale of their printability considering their susceptibility to thermal distortion. For alloys that are highly susceptible, appropriate AM variables like laser power, layer thickness and scanning speed need to be adjusted based on equation (7) to reduce thermal strain and distortion.

### Composition change

At high temperatures encountered during AM, significant vaporization of alloying elements occur from the molten pool. Since some alloying elements are more volatile than others, selective vaporization of alloying elements often results in a significant change in the composition of the alloy. For example, during laser welding of aluminum alloys, losses of magnesium and zinc result in pronounced changes of their concentrations. The composition change, in turn causes degradation of hardness, corrosion resistance and tensile properties.

The vaporization fluxes of alloying elements, *J_i,_* can be estimated from the Langmuir equation^8^:


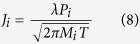


where *P*_*i*_ is vapor pressure over the alloy, *M*_*i*_is the molecular weight of element *i*, *T* is temperature and *λ* is a positive fraction accounting for the condensation of some vaporized atoms. Temperatures of the molten pool are calculated using a three-dimensional heat transfer and fluid flow model and the equilibrium vapor pressures of all alloying elements are estimated from available thermodynamic data. The amount of material vaporized, 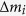, can be estimated as


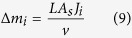


where *v* is scanning speed, *L* is the track length and *A*_*s*_ is surface area of the molten pool. The volume of material *V* deposited can be approximated by


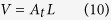


where *A*_*t*_ is transverse cross sectional area of the molten pool perpendicular to the scanning direction at the point of highest depth. The weight percentage of element *i* after vaporization, *W*_*f*_, can be calculated using


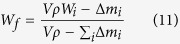


where *W*_*i*_ is the initial weight percentage of element *i* in the powder. The composition change is the difference in weight percentages of element *i* in the powder and deposited material.

**Figure 4:**
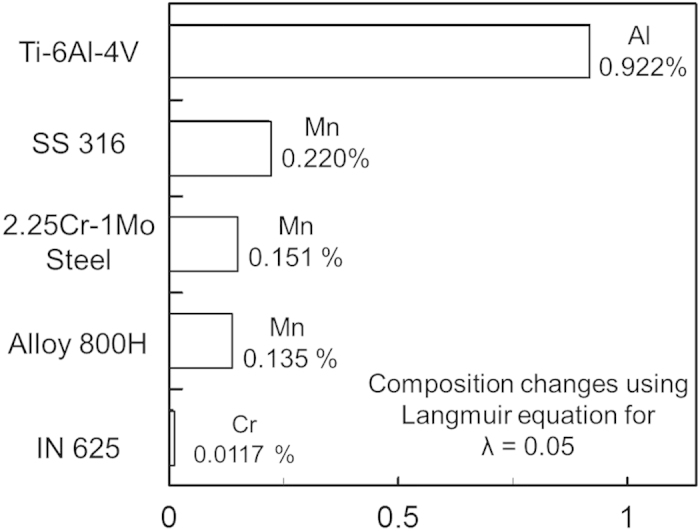
Composition change in wt% of the most volatile elements due to vaporization for five alloys using Parameter Set 3 in Table 1.

Figure 4 shows the most volatile alloying elements to be manganese in 2.25Cr-1Mo steel, Alloy 800H and SS 316, chromium in IN 625, and aluminum in Ti-6Al-4V. Figure 4 shows that Ti-6Al-4V is the most susceptible and IN 625 is the least susceptible to change in composition, respectively.

Results from Brice *et al.*^23^ show an average composition change of about 0.9 wt% Al for electron beam deposition of Ti-6Al-4V. Temperatures of the molten pool are higher for Ti-6Al-4V than the other alloys for the same heat input per unit length due to its relatively low thermal conductivity and density. High temperatures and the high equilibrium vapor pressure of aluminum result in a larger composition change for Ti-6Al-4V compared to the other alloys for identical process parameters.

A scale has been developed to rank the printability of common alloys by considering composition change. Figure 4 shows that IN 625 will experience the smallest composition change and Ti-6Al-4V will experience the largest. Therefore, IN 625 and Ti-6Al-4V will be the least and most susceptible to composition change among the alloys considered. For alloys highly susceptible to composition change, care should be taken to adjust appropriate AM variables such as laser power density and scanning speed to reduce loss of volatile alloying elements.

### Lack of fusion defects

Lack of fusion is caused by inadequate penetration of the molten pool of an upper layer into either the substrate or the previously deposited layer. Inadequate penetration can cause voids to form in the final product which are typically larger than 10 micrometers in equivalent diameter. These voids can also be generated by gas entrapment during the atomization of the powder particles and the shape of the deposit. For example, omega shape deposits create much more inter layer porosity compared to lens shape deposits^24^. These macro-pores are much larger than the micro-pores caused by surface moisture absorption, oxidation and dissolved gases. Penetration depth depends on the physical properties of the alloy powder and processing conditions like laser power, scanning speed and deposition strategy. However, various alloys exhibit different depths of penetration for identical AM processing conditions depending on their thermo-physical properties. Therefore, alloys differ in their susceptibilities to lack of fusion defects.

Adequate fusion and inter-layer bonding for different alloys can be examined by considering a non-dimensional lack of fusion index, *LF*, defined by


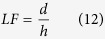


where *d* is the penetration depth of the molten pool and *h* is the thickness of a layer of material deposited onto the substrate or previously deposited layer. In order for a deposited layer to bond properly with a previous layer, the penetration depth of the molten pool, *d*, should exceed the layer thickness, *h*, and adequately remelt the previously deposited layer. The minimum possible value of LF for establishing contact between two successive layers is 1, indicating a penetration depth (*d*) equal to layer thickness (*h*). However, this contact is inadequate for good bonding. Carroll *et al.*^25^ reported a 99.999% dense part for direct energy deposition AM of Ti-6Al-4V, indicating proper inter-layer bonding. A corresponding LF index was estimated as 1.15. So, a penetration of 15% of the layer thickness into the previous layer signifies good interlayer bonding.

**Figure 5:**
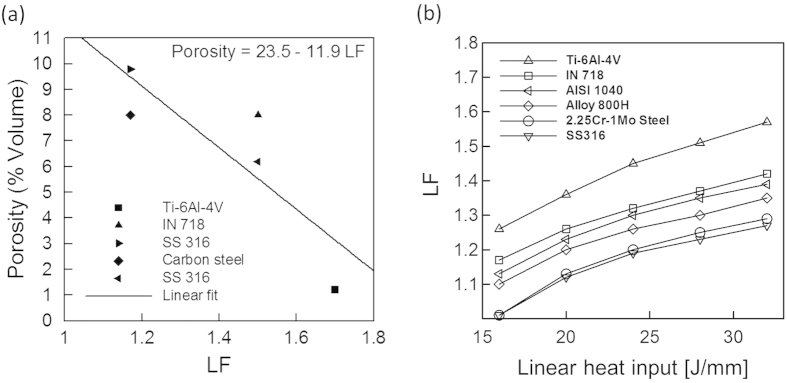
Correlation between LF and (a) macro-porosity for Ti-6Al-4V^26^, IN 718^27^, SS 316^28^, carbon steel^29^ and SS 316^30^ and (b) linear heat input for six different alloys with a constant layer thickness of 0.38 mm.

The three-dimensional heat transfer and fluid flow model is used to estimate penetration depths for six alloys over a range of linear heat inputs. Figure 1(c) shows the computed molten pool for Ti-6Al-4V, as an example, and similar results for the other five alloys are shown in the supplementary document. Figure 5(a) shows an inverse relationship between the macro-porosity resulting from lack of fusion defects^26^,^27^,^28^,^29^,^30^ and the corresponding estimated LF values. For larger values of LF, the molten pool penetrates deeper into the previously deposited layer to provide adequate inter-layer bonding. Figure 5(b) shows the susceptibilities of various alloys to lack of fusion defects are unaffected by the heat input. Also, Fig. 5(b) shows that for a given heat input, Ti-6Al-4V will have the highest value of LF while SS 316 will have the lowest. Therefore, Ti-6Al-4V and SS 316 are the least and most susceptible to lack of fusion defects, respectively, among the alloys considered. For alloys that are highly susceptible to lack of fusion defects, AM variables like laser power, scanning speed and powder feed rate should be appropriately adjusted to attain an adequate depth of penetration.

## Conclusion

In summary, relative susceptibilities of various alloys to thermal distortion, loss of alloying elements and lack of fusion defects that determine their printability have been examined quantitatively and validated with independent experimental data. Results show that Ti-6Al-4V is most susceptible to thermal strain and distortion during AM compared to IN 625 and SS 316. Ti-6Al-4V and IN 625 are the most and least susceptible to composition change, respectively. The computed lack of fusion index shows that SS 316 and Ti-6Al-4V have the highest and lowest vulnerability to lack of fusion defects, respectively. The results provide an understanding of the printability of various powder materials based on their physical properties and how they would behave under commonly used process conditions in AM.

## Methods

A computer code that solves the equations of conservation of mass, momentum and energy has been developed to calculate transient temperature and velocity fields and the geometry of the molten pool. The governing equations are discretized by following a control volume method and solved using tri-diagonal matrix algorithm (TDMA) with appropriate boundary conditions and temperature dependent material properties. The material database for thermo-physical and chemical properties of the alloys are created using JMatPro® V8 software. The three components of velocity and enthalpy are iterated at each time step. The total computational time depended on the part dimensions, process conditions and the alloy. For example, for the deposition of a 1 cm long slab of Ti-6Al-4V using 141,075 grid points and the data set 1 in Table 1, 6 minutes and 48 seconds was needed in a personal computer with 3.20 GHz Intel Pentium 4 processor and 2 GB RAM. Typically a total of about 5 billion linear equations need to be solved cumulatively for all time steps for each layer.

## Additional Information

**How to cite this article**: Mukherjee, T. *et al.* Printability of alloys for additive manufacturing. *Sci. Rep.* **6**, 19717; doi: 10.1038/srep19717 (2016).

## Supplementary Material

Supplementary Information

## Figures and Tables

**Table 1 t1:** Process conditions used in numerical calculations.

Parameter Set	Laser power (W)	Beam radius (mm)	Scanning speed (mm/s)	Layer thickness (mm)	Substrate thickness (mm)
1	190	0.5	12.5	0.38	4
2	600	0.5	7.5	0.25	7
3	1000	0.5	12.5	0.38	4

**Table 2:** Variables used in dimensional analysis in the MLTθ system.

Variable	Dimension
Volumetric thermal expansion coefficient, *β*	θ^−1^
Temperature gradient, Δ*T* = *T*_*P*_*−T*_*S*_, where *T*_*P*_ and *T*_*S*_ refer respectively to peak and surrounding temperature	θ
Deposition layer thickness, *h*	L
Thermal diffusivity, 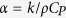 , where *k*, *ρ* and *C*_*P*_ are thermal conductivity, density and specific heat, respectively of deposit material	L^2^T^−1^
Heat input per unit length, 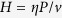 , where *η*, *P*, and *v* refer to absorption coefficient, beam power and scanning speed, respectively	MLT^−2^
Melt pool volume, *V*	L^3^
Flexural rigidity of the substrate plate, *EI*, where *E* and *I* refer respectively to elastic modulus and second moment of inertia	ML^3^T^−2^
Thermal strain parameter, ε^*^	M^0^L^0^T^0^θ^0^
