# Supplementary material for: Printability of alloys for additive manufacturing
Source: Sci Rep. 2016 Jan 22;6:19717. doi: 10.1038/srep19717 (PMC4726422; doi:10.1038/srep19717)
Supplement: Supplementary Information [file srep19717-s1.pdf]

Printability of alloys for additive manufacturing

T. Mukherjee, J. S. Zuback, A. De and T. DebRoy*

Department of Materials Science and Engineering

The Pennsylvania State University

University Park, Pennsylvania, 16802, USA

*Corresponding author: debroy@psu.edu (T.D.)

Supplementary Information includes:

Supplementary Discussions 1 - 3

Supplementary Figures 1 - 3

Supplementary Tables 1 – 6

Supplementary Equations 1 – 3

Supplementary References

Supplementary Discussion 1 | Calculation of thermal strain parameter for Ti-6Al-4V

The thermal strain parameter (ε^*) is calculated using equation (1) of the main document. The variables required for the calculations are taken from the literature and are listed in Supplementary Table 1.

Supplementary Table 1. Parameters used for thermal strain parameter calculation

Parameters	Values
Laser power (W)	4000
Laser beam radius (mm)	2
Laser scanning speed (mm/s)	10.6
Layer thickness (mm)	0.89
Ambient Temperature (K)	298
Liquidus Temperature (K)	1928
Total length (mm)	152.4
Substrate width (mm)	38.1
Substrate thickness (mm)	12.7
Track length (mm)	101.6

For equation (1) of the main paper, ΔT is calculated as the difference of liquidus temperature and surrounding temperature which is considered as the ambient temperature. Linear heat input (*H*) is estimated from laser power and scanning speed. Total time (*t*) is represented as the time taken by the laser beam to completely travel the length of the track. Therefore, the total time is the total track length divided by the scanning speed. The area moment of inertia of the substrate plate (*I*) is calculated based on the substrate width and thickness considering a rectangular cross-section. Fourier number (*F*) depends on scanning speed, pool length and thermal diffusivity. The pool length is calculated using the numerical model of AM process described in the Supplementary Discussion 4. Numerical modeling is done using the parameters mentioned in the Supplementary Table 1 and thermos-physical properties of Ti-6Al-4V given in the Supplementary Table 2.

The estimated value of thermal strain parameter (ε^*) from equation (1) is 1.5×10^{-3}. On the other hand, the thermal strain (ε) extracted from the reported value of experimentally measured maximum distortion is 3.3×10^{-3}. So, the coordinate of the point corresponding to Ti-6Al-4V in Figure 1(a) of the main paper is (1.5×10^{-3}, 3.3×10^{-3}).

Supplementary Table 2 | Thermo-physical properties for Ti-6Al-4V[1]

Thermo-physical properties	Values
Density (kg/m^3)	4000
Solidus Temperature (K)	1878
Liquidus Temperature (K)	1928
Thermal conductivity (W/m K)	$1.57+ 1.61\times 10^{-2}$ T – 1.00×10^{-6} T^2
Specific heat (J/kg K)	$492.6+ 0.25$ T – 0.418×10^{-4} T^2
Latent heat of fusion (J/kg)	2.84×10^5
Coefficient of thermal expansion (/K)	0.86×10^{-5}
Viscosity of liquid alloy (kg/m s)	7.0×10^{-3}

Error analysis in thermal strain calculation

From equation (7) of the main article we obtain,

$$\varepsilon = \frac{0.9081\ \beta\Delta T}{EI}\frac{t}{F\sqrt{\rho}}H^{3/2} + 0.0009$$

Assuming a flat molten pool surface, the error in the estimation of molten pool length will be 3%. We can rewrite equation (7) as:

$$\varepsilon = K\,\omega + 0.0009$$

Where, K is the constant term, ω is pool length and ΔT is the temperature difference. Assuming a flat molten pool surface the calculated strain will be,

$$\varepsilon' = K(1.03\omega) + 0.0009$$

So, the absolute error in this calculation will be,

$$\varepsilon' - \varepsilon = 0.03K\,\omega$$

Therefore, the percentage error in the thermal strain calculation will be,

$$\%\ \text{error} = \frac{\varepsilon' - \varepsilon}{\varepsilon}\times 100\% \approx \frac{0.03K\,\omega}{K\,\omega}\times 100\% = 3.0\%$$

Supplementary Discussion 2 | Equilibrium Vapor Pressure Calculations

Supplementary Table 3 shows that power densities used in AM are typically lower than, but comparable to, those used in laser welding. The resulting higher peak temperatures in laser welding yield higher evaporative mass loss per unit time compared to AM. However, composition change is highly dependent on the surface area to volume ratio. Although the

vaporization rates during laser welding are higher, the larger weld pool volumes result in lower surface area to volume ratios compared to AM. The small molten pool volumes, and consequently large surface to volume ratios of the molten pools in AM are anticipated to make parts susceptible to pronounced composition change.

Supplementary Table 3 | Power densities reported in AM and laser welding literature

Process	Power density [W/mm2]	Reference
Direct energy deposition AM	3057-4586	[2]
Selective laser melting AM	4076	[3]
Electron beam melting AM	2389	[4]
Laser welding	1000-7000	[5]
Laser welding	2149-14154	[6]
Laser welding	5167	[7]

Calculations of equilibrium vapor pressure of elements over their pure liquid were made using the data presented in Supplementary Table 4 and Supplementary Table 5 to examine the printability of alloys in terms of vaporization. The coefficients in Supplementary Table 4 correspond to the equation from Gale[8] given by

$$\log(P_i^0 \times 760) = -\frac{A}{T} + B + C\log T + 10^{-3}\,DT \qquad (1)$$

while the coefficients in Supplementary Table 5 correspond to the equation from Yaws[9] and are given by

$$\log(P_i^0 \times 760) = A + \frac{B}{T} + C\log T + DT + ET^2 \qquad (2)$$

In both equations (14) and (15), P_i^0 is expressed in [atm] and T is expressed in [K].

Supplementary Table 4 | Coefficients used for calculating equilibrium vapor pressure

Element	A (x10^4)	B	C	D
Al	1.6450	12.36	–1.023	0
Cu	1.765	13.39	–1.273	0
Mg	0.7550	12.79	–1.41	0
Ni	2.24	16.95	–2.01	0
Si	2.09	10.84	–0.565	0
Ti	2.32	11.74	–0.66	0

Supplementary Table 5 | Coefficients used for calculating equilibrium vapor pressure

Element	A	B (x10^4)	C	D	E
Cr	–80.3456	–1.2221	2.9746	–6.84x10^{-3}	5.2454x10^{-7}
Fe	11.5549	–1.9538	–0.62549	–2.7182x10^{-9}	1.9086x10^{-13}
Mo	74.9735	–4.1955	–20.072	3.2166x10^{-3}	–2.2507x10^{-7}
Nb	–64.3485	–2.9438	23.622	–3.9155x10^{-3}	2.0660x10^{-7}
V	52.0677	–3.1989	–12.620	1.6179x10^{-3}	–1.0505x10^{-7}

Calculations from equations (1) and (2) correspond to the equilibrium vapor pressure of elements over the pure liquid metal. Considering that the liquid alloy behaves ideally near its boiling point, the equilibrium vapor pressure P_i of element i over the liquid alloy is estimated as

$$P_i = X_i \, P_i^0 \qquad (3)$$

where X_i and P_i^0 are the mole fraction and the equilibrium vapor pressure of element i over its pure liquid, respectively. The compositions of the alloys shown in Supplementary Table 6 are used to calculate the mole fractions of each element. Supplementary Figure 1 shows the equilibrium vapor pressure of the constituting elements over the liquid alloy for five different alloys as a function of temperature. The plot shows that the vapor pressure of the elements has a strong dependence on temperature. The vaporization flux J_i is estimated from the Langmuir equation and the composition change for various elements is calculated as described in the main document.

Supplementary Table 6 | *Alloy compositions*

	Wt% of element									
Alloy	Al	Cr	Cu	Fe	Mn	Mo	Nb	Ni	Si	Ti	V
IN 625	0.2	21.5	-	4	-	9	3.6	61.5	-	0.2	-
SS 316	-	17	-	68	2	-	-	12	1	-	-
800H	0.6	21	0.75	39.5	1.5	-	-	34.95	1	0.6	-
2.25Cr-1Mo Steel	0.6	2.25	-	95.61	0.5	1	-	0.045	0.5	0.6	-
Ti-6Al-4V	6	-	-	-	-	-	-	-	-	90	4

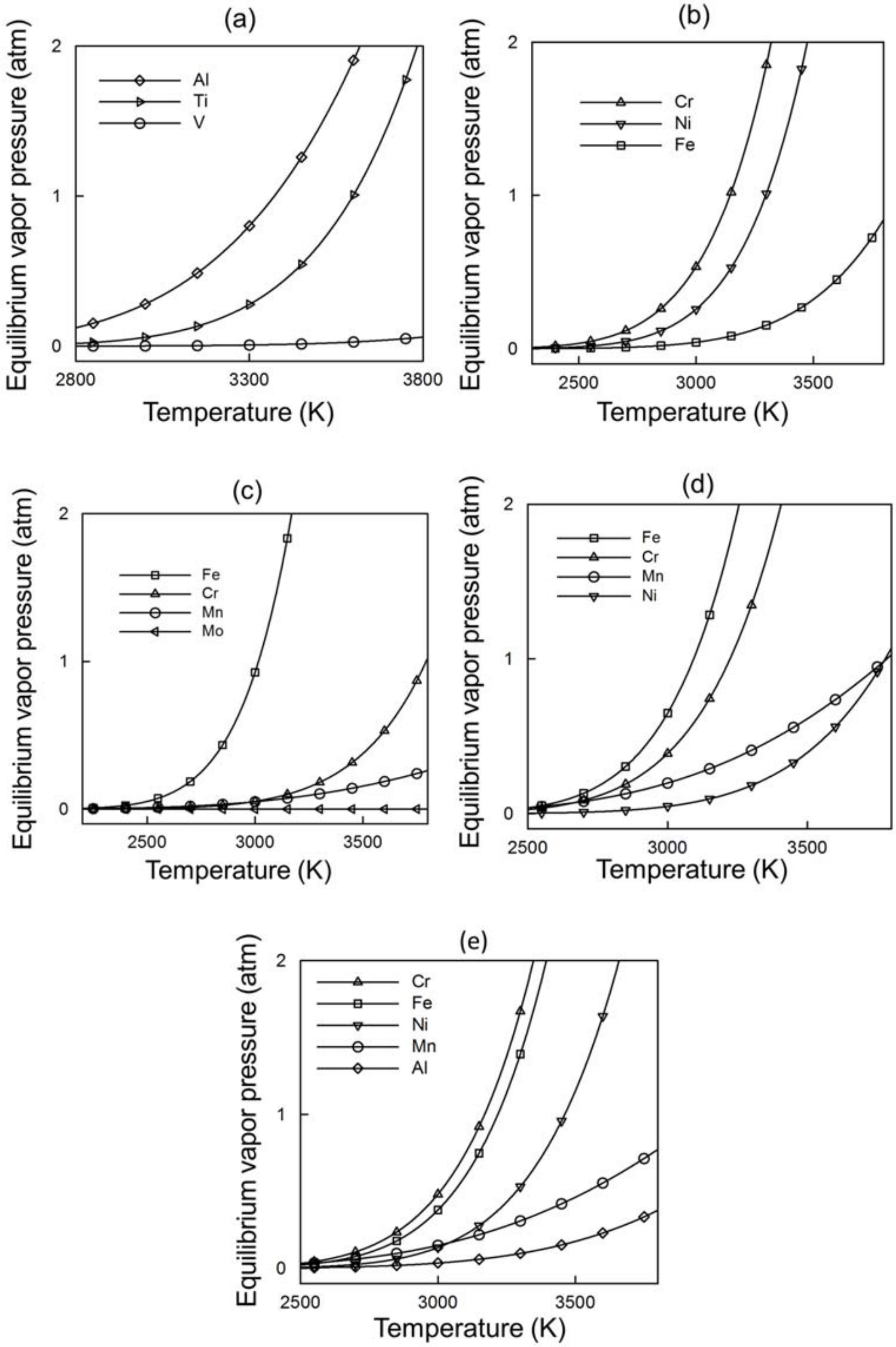

Supplementary Figure 1 | Equilibrium vapor pressures for alloying elements of different alloys as functions of temperature. (a) Ti-6Al-4V (b) IN625 (c) 2.25Cr-1Mo Steel (d) SS 316 (e) Alloy 800H

Supplementary Discussion 3. Numerical model to calculate pool dimension in AM

Laser beam assisted AM produces near net shape products in a layer-by-layer deposition, as shown in Supplementary Figure 2, where the path of laser beam movement and alloy powder deposition are calculated directly from the digital geometry of the product[10-13]. For direct energy deposition process, the powder flows from the nozzle coaxially with the laser. Part of the energy from laser is absorbed by the powder particle in flight from the nozzle to the substrate. The remaining energy melts the powder particles. During the process, the alloy powder undergoes rapid heating, melting, solidification and cooling, subsequently.

A comprehensive heat transfer and fluid flow numerical model is employed to determine the dimensions of the molten pool during the AM process. The model calculates transient three-dimensional temperature and velocity fields from process variables, such as laser power, power density distribution, scanning speed, chemical composition, particle size, feed rate, and thermos-physical properties of the alloy powder[10]. The model solves the three-dimensional conservation equations for mass, momentum, and energy. These equations are available in standard text books[14] as well as existing literature[15]. The thermo-physical properties used for calculations are shown in Supplementary Table 2 for Ti-6Al-4V, as an example. The transient heat transfer and fluid flow calculations are performed for a rectangular solution domain representing the substrate, deposited layers, and the surrounding gas as shown in Supplementary Figure 2. A control volume method is implemented to discretize the governing equations by dividing the solution domain into small rectangular control volumes. The continuous movement of laser beam is discretized using small shifts of the laser beam. The time-step for each shift of the laser beam is calculated from the deposition length and the laser scanning speed. For each shift of the laser beam, a set of cells under the beam are assigned properties of the powder material. An idle time is considered at the end of the simulation of each layer. The deposition of a new layer is started from the initial location on the top of the previously deposited layer. The procedure is repeated until the simulation of all the layers is completed. At the end of the simulation of all layers sufficient cooling time is also added.

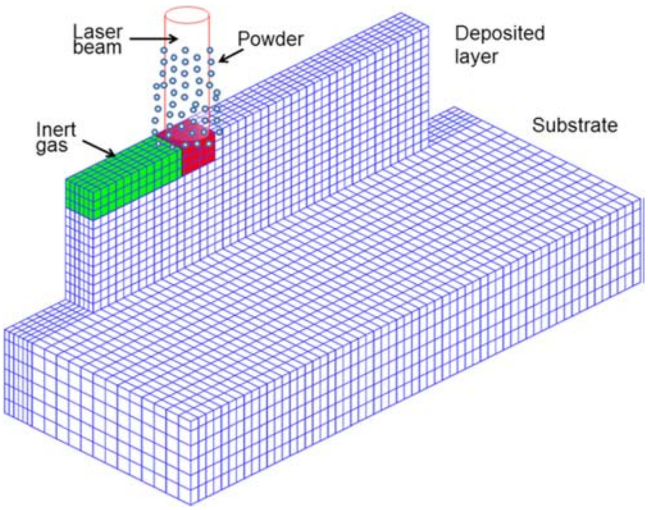

Supplementary Figure 2 | Schematic representation of laser assisted AM process[10]. Illustration of the process showing the processing of alloy powder by the laser beam to produce the deposited layer on substrate surface.

Supplementary Figure 3 shows the computed melt pool geometry for the second layer deposited. Each color band in the profile represents a temperature range shown in the legend. The yellow colored regions in all the figures indicate that the deposited material has reached the solidus temperature of the corresponding alloy. The red zone indicates the molten pool for the alloy. The vectors show the computed velocity fields in the molten region. A reference vector is shown by an arrow and a comparison of the length of this arrow with the vectors shown in the plots reveals the magnitudes of the computed velocities. The depth of the molten pool is calculated by measuring the distance from the top to the bottom of the molten pool. This depth of penetration is utilized to calculate LF as described in the main document.

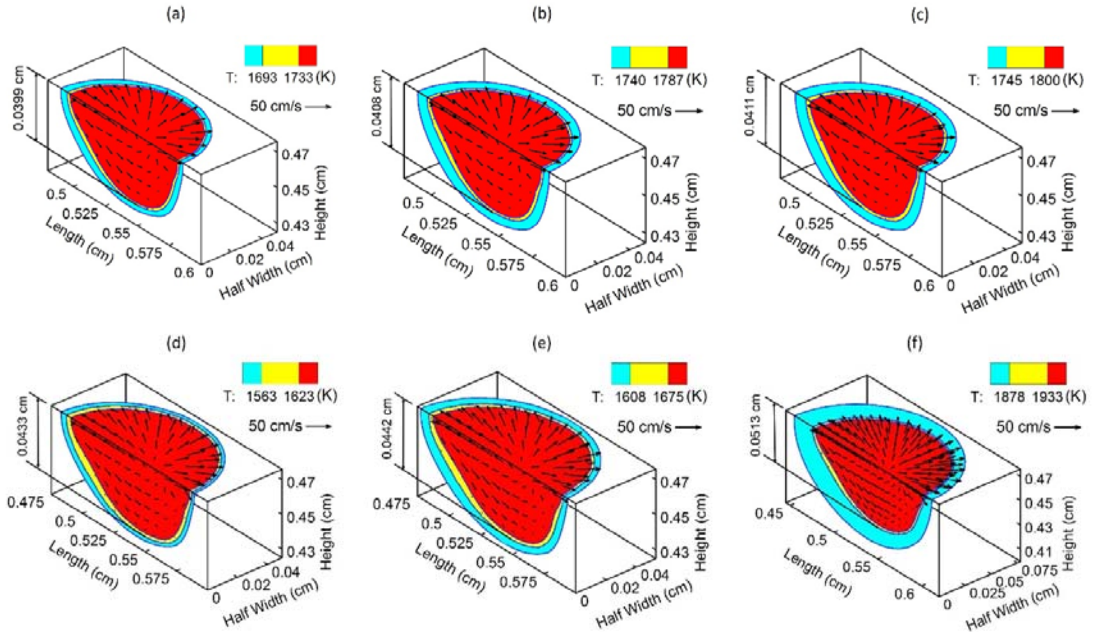

Supplementary Figure 3 | Calculation of depth of fusion of molten pool for different alloys for the processing conditions presented in Parameter Set 1 in Table 1 of the main document. (a) Stainless steel SS 316 (b) 2.25Cr-1Mo steel (c) AISI 1040 steel (d) Inconel IN 718 (e) Alloy 800 H (d) Titanium alloy Ti6Al4V

Supplementary References

[1] Mills, K.C. *Recommended Values of Thermophysical Properties for Selected Commercial Alloys*. (Woodhead publishing, Cambridge, 2002).

[2] Dinda, G. P., Dasgupta, A. K. & Mazumder, J. *Mater. Sci. Eng. A.* **509,** 98-104 (2009).

[3] Vilaro, T., Colin, C., Bartout, J. D., Naze, L. & Sennour, M. *Mater. Sci. Eng. A.* **534,** 446-451 (2012).

[4] Klassen, A., Scharowsky, T. & Korner, C. *J. Phys. D Appl. Phys.* **47,** 275303 (2014).

[5] He, X., DebRoy, T. & Fuerschbach, P. W. *J. Appl. Phys.* **96,** 4547-4555 (2004).

[6] Mundra, K. & DebRoy, T. *Metall. Trans. B.* **24B,** 145-155 (1993).

[7] Zhao, H. & DebRoy, T. *Metall. Mater. Trans. B.* **32B,** 163-172 (2001).

[8] Gale, W.F. & Totemeier, T.C. *Smithells Metals Reference Book*, 8th edition. (Butterworth-Heinemann, Burlington, 2003)

[9] Yaws, C.L. *Handbook of Vapor Pressure* (Gulf Pub. Co, Houston, 1994).

[10] Manvatkar, V., De, A. & DebRoy, T. Heat transfer and material flow during laser assisted multi-layer additive manufacturing. *J. Appl. Phys.* **116**, 1 (2014).

[11] Mercelis, P. & Kruth, J. P. Residual stresses in selective laser sintering and selective laser melting. *Rapid Prototyping J*. **12,** 254 (2006).

[12] Manvatkar, V., De, A., & DebRoy, T. Spatial variation of melt pool geometry, peak temperature and solidification during laser assisted additive manufacturing process. *Mater. Sci. Tech. Ser.* **31,** 924 (2015).

[13] Denlinger, E. R., Heigel, J. C., Michaleris, P., & Palmer, T. A. Effect of inter-layer dwell time on distortion and residual stress in additive manufacturing of titanium and nickel alloys. *J. Mater. Process. Tech.* **215,** 123 (2015).

[14] Patankar, S. V. Numerical Heat Transfer and Fluid Flow (McGraw-Hill, New York, 1982)

[15] DebRoy, T. & David, S.A. Physical processes in fusion welding. *Rev. Mod. Phys*. **67(1),** 85-112 (1995).
